# Compromised mammillary body connectivity and psychotic symptoms in mice with di- and mesencephalic ablation of ST8SIA2

**DOI:** 10.1038/s41398-022-01816-1

**Published:** 2022-02-03

**Authors:** Melike Küçükerden, Ute E. Schuster, Iris Röckle, Gonzalo Alvarez-Bolado, Kerstin Schwabe, Herbert Hildebrandt

**Affiliations:** 1 Institute of Clinical Biochemistry, Hannover Medical School, Hannover, Germany; 2 Center for Systems Neuroscience Hannover (ZSN), Hannover, Germany; 3 Institute for Anatomy and Cell Biology, Ruprecht-Karls-University Heidelberg, Heidelberg, Germany; 4 Department of Neurosurgery, Hannover Medical School, Hannover, Germany

**Keywords:** Molecular neuroscience, Schizophrenia

## Abstract

Altered long-range connectivity is a common finding across neurodevelopmental psychiatric disorders, but causes and consequences are not well understood. Genetic variation in *ST8SIA2* has been associated with schizophrenia, autism, and bipolar disorder, and *St8sia2*^*−/−*^ mice show a number of related neurodevelopmental and behavioral phenotypes. In the present study, we use conditional knockout (cKO) to dissect neurodevelopmental defects and behavioral consequences of *St8sia2* deficiency in cortical interneurons, their cortical environment, or in the di- and mesencephalon. Neither separate nor combined cortical and diencephalic ablation of *St8sia2* caused the disturbed thalamus-cortex connectivity observed in *St8sia2*^*−/−*^ mice. However, cortical ablation reproduced hypoplasia of corpus callosum and fornix and mice with di- and mesencephalic ablation displayed smaller mammillary bodies with a prominent loss of parvalbumin-positive projection neurons and size reductions of the mammillothalamic tract. In addition, the mammillotegmental tract and the mammillary peduncle, forming the reciprocal connections between mammillary bodies and Gudden’s tegmental nuclei, as well as the size of Gudden’s ventral tegmental nucleus were affected. Only mice with these mammillary deficits displayed enhanced MK-801-induced locomotor activity, exacerbated impairment of prepulse inhibition in response to apomorphine, and hypoanxiety in the elevated plus maze. We therefore propose that compromised mammillary body connectivity, independent from hippocampal input, leads to these psychotic-like responses of *St8sia2*-deficient mice.

## Introduction

Broad evidence links genetic risk factors with common neurodevelopmental predispositions to psychiatric disorders such as schizophrenia [1], autism spectrum and bipolar disorders [2, 3], and, arguably, depression [4]. To gain mechanistic insights into the etiology of these diseases, numerous studies focus on the impact on synaptic connectivity and local network changes. In contrast, the causes and consequences of alterations in structural and functional long-range connectivity, although frequently reported for psychiatric and other brain diseases [5–8], receive considerably less attention.

*ST8SIA2* is a neurodevelopmental gene that has been repeatedly associated with schizophrenia [9–12], but also with autism [13, 14], bipolar disorder [12, 15–17], and depression [18]. Loss of *St8sia2* in mice by conventional knockout leads to several neuropathological traits with links to psychiatric disorders. Among others, *St8sia2*^*−/−*^ mice show reduced densities of parvalbumin-positive interneurons in the prefrontal cortex [19, 20]. They also develop enlarged lateral ventricles and size reductions of the thalamus, accompanied by a smaller internal capsule, a highly disorganized pattern of fibers connecting thalamus and cortex, and compromised glutamatergic thalamocortical input [21]. Furthermore, ectopic synapse formation and reduction of basal synaptic transmission in the hippocampus, as well as impaired glutamatergic transmission and synaptic plasticity in the amygdala have been observed [22–24]. Individually or combined, these changes may give rise to the cognitive deficits and altered behaviors of *St8sia2*^*−/−*^ mice, including impaired working memory and fear learning, deficits in sensorimotor gating, reduced anxiety, increased exploratory and locomotor activity, or reduced social interactions with increased aggression [21–23, 25, 26]. As shown recently, increased aggression and impaired fear learning could be reproduced by local silencing of *St8sia2* in the early postnatal amygdala and were assigned to altered glutamatergic synaptic transmission [23]. Both could be normalized by administration of the partial NMDA-receptor agonist D-cycloserine to the amygdala. In contrast, the local silencing in the amygdala had no effect on anxiety traits and intracerebroventricular delivery but no local application of D-cycloserine was efficient in normalizing anxiety levels, indicating an involvement of glutamatergic deficits in other brain regions and circuits. Based on the neurodevelopmental phenotypes of *St8sia2*^*­−/−*^ mice, reduced synaptic transmission in the hippocampus or impaired glutamatergic thalamocortical input on inter- and/or projection neurons of the prefrontal cortex would be plausible candidates.

ST8SIA2 is a glycosyltransferase, which assembles the polymeric glycan polysialic acid (polySia) on NCAM [27]. In contrast to the second polysialyltransferase, ST8SIA4, which is mainly implicated in synaptic transmission and plasticity, ST8SIA2 is rapidly down-regulated after birth and absent from most parts of the adult brain [28]. The complete loss of polySia in *St8sia2*;*St8sia4* double knockout mice leads to postnatal growth retardation and precocious death, as well as to defects of brain development that are partially recapitulated in *St8sia2*-deficient mice, as described above. Another neurodevelopmental phenotype of polySia-negative mice is a marked hypoplasia of the mammillothalamic tract (mt) [29], which has not yet been explored in *St8sia2*^*−/−*^ animals. As part of the Papez circuit, the mt connects the mammillary bodies (MB) of the hypothalamus to the anterior thalamic nuclei. Although largely neglected, there is evidence that severely compromised MB connectivity is linked to a number of behavioral changes including hyperactivity, hypoanxiety, and deficits of spatial working memory [30–36].

Here, we used mice with conditional knockout (cKO) of *St8sia2* in the cortex (*Emx1-Cre;St8sia2*^*f/f*^), the diencephalon and brainstem (*Foxb1-Cre;St8sia2*^*f/f*^), or both (*Foxb1-Cre; Emx1-Cre;St8sia2*^*f/f*^), to analyze consequences for thalamus-cortex or MB connectivity, as well as mice with an interneuron-specific deletion of *St8sia2* (*Lhx6-Cre;St8sia2*^*f/f*^) that recapitulate the reductions of parvalbumin-positive cortical interneurons in *St8sia2*^*−/−*^ mice [20]. None of the analyzed cKO strains displayed the thalamocortical deficits of *St8sia2*^*−/−*^ mice, but distinct aspects of impaired MB connectivity were detected in *Foxb1-Cre;St8sia2*^*f/f*^ as compared to *Emx1-Cre;St8sia2*^*f/f*^ animals and allowed us to assess their contributions to psychosis-related behaviors.

## Materials and methods

See Supplementary Material for details.

### Mice

Mice were bred in the central animal facility of Hannover Medical School. Experimental procedures were conducted in accordance with the German Animal Welfare Act and approved by the local authorities (Niedersächsisches Landesamt für Verbraucherschutz und Lebensmittelsicherheit, permission nos. 33.12-42502-04-15/1902 and -18/2932).

*St8sia2*^*−/−*^ mice, *St8sia2*^*f/f*^ mice with loxP flanked (“floxed”) *St8sia2* alleles, *Lhx6-Cre;St8sia2*^*f/f*^, and *Emx1-Cre;St8sia2*^*f/f*^ mice were generated as described previously [20]. In addition, *St8sia2*^*f/f*^ mice were crossed with mice expressing Cre-recombinase under the diencephalon- and brainstem-specific *Foxb1* promoter [37] (*Foxb1-Cre;St8sia2*^*f/f*^) and these mice were cross-bred with *Emx-1-Cre;St8sia2*^*f/f*^ mice to obtain *Foxb1-Cre;Emx-1-Cre;St8sia2*^*f/f*^ double mutant mice. Based on the routine inspections, all of the cKO lines appeared healthy and showed no overt alterations in behavior and activity. As determined for the male mice of cohort 2 prior to behavioral testing (see below), all cKO mice were of comparable body weights, while *St8sia2*^*−/−*^ mice showed a slight reduction (Supplementary Table 1).

### Brain sectioning and morphological assessment

Three months old male mice were perfused, 50 µm vibratome sections were generated and morphometric evaluation of coded and randomized images of unstained brain sections was performed by a blinded observer as described earlier [29, 38]. Bregma levels were determined based on Paxinos and Franklin, 2001 [39]. Mean values of the measurements in both hemispheres were averaged for each animal.

### Immunofluorescence staining and evaluation

Antibodies (with vendor, catalog number, and RRID), staining, and evaluation procedures are detailed in Supplementary Methods.

### Behavioral testing

Behavioral assessments were carried out with two separate cohorts of 3–6-month-old male mice. Cohort 1 consisted of *St8sia2*^*f/f*^, *St8sia2*^*−/−*^, *Lhx6-Cre;St8sia2*^*f/f*^*, Foxb1-Cre;St8sia2*^*f/f*^, and *Emx1-Cre;St8sia*^*f/f*^ mice. In addition to these lines, *Foxb1-Cre;Emx1-Cre;St8sia2*^*f/f*^ mice were included in cohort 2. As detailed in Supplementary Materials, mice in cohort 1 were tested in the open field and a delayed nonmatch-to-place T-maze task. Mice in cohort 2 were tested under different conditions in the open field, for prepulse inhibition of the acoustic startle response (PPI), in the elevated plus maze, in a dark-light box, and for marble burying. As detailed in Supplementary Methods, open field, elevated plus maze, and dark-light box experiments were analyzed by automated video tracking, PPI was performed in automated Startle Response System chambers, and marble burying was scored on randomized photographs by a blinded observer. T-maze experiments were scored without blinding or randomization. For technical reasons, less than five *Foxb1-Cre;Emx1-Cre;St8sia2*^*f/f*^ could be evaluated over the entire test period in the dark-light box. Therefore, this group was excluded from statistical evaluation.

### Statistics

Statistical analyses, including tests of normality and equal variances, were performed using Prism 8.0 (GraphPad, San Diego, CA, USA) as specified in the Supplementary Methods. Based on previous experience [21], a group size of five was considered the minimal requirement for statistical evaluation. Statistical test results together with the exact sample sizes are reported in the respective figure legends.

## Results

### Mice with cKO of *St8sia2* in the di- and mesencephalon exclusively reproduce mammillary body deficits of *St8sia2*^*−/−*^ mice

Interneuron- and cortex-specific recombination of *St8sia2* in *Lhx6-Cre;St8sia2*^*f/f*^ and *Emx1-Cre;St8sia2*^*f/f*^ mice has been characterized before [20]. To validate the conditional recombination of *St8sia2* in *Foxb1-Cre;St8sia2*^*f/f*^ mice, the presence of the recombined allele was confirmed by genomic PCR, while qPCR analysis revealed a significant reduction of wild-type mRNA and identified transcripts of the recombined allele in isolated thalamic tissue of E13.5 and E14.5 *Foxb1-Cre;St8sia2*^*f/f*^ embryos, respectively (Supplementary Fig. 1).

**Fig. 1:**
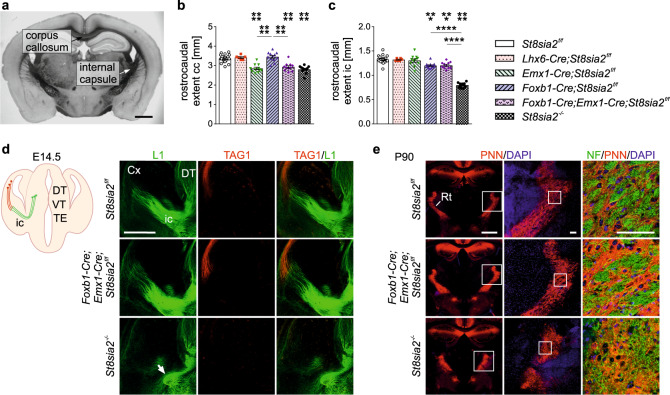
Deficits of thalamus-cortex connectivity in *St8sia2*^*−/−*^ mice are not reproduced by any of the cKO lines. **a** Representative image showing the corpus callosum and the internal capsule on a coronal brain section from a *St8sia2*^*f/f*^ mouse. **b**, **c** Rostrocaudal extent of the corpus callosum (cc) and the internal capsule (ic) for the indicated genotypes. Graphs show means ± SEM and individual data points of *n* = 24, 6, 11, 12, 11, and 11 *St8sia2*^*f/f*^, *Lhx6-Cre;St8sia2*^*f/f*^, *Emx1-Cre;St8sia2*^*f/f*^, *Foxb1-Cre;St8sia2*^*f/f*^, *Foxb1-Cre;Emx1-Cre;St8sia2*^*f/f*^ and *St8sia2*^*−/−*^ mice, respectively. One-way ANOVA indicated significant differences (*p* < 0.0001; F_5,69_ = 27.28 in **b**, F_5,69_ = 88.33 in **c**). Holm–Sidak’s post hoc tests were applied and significant differences for comparisons with *St8sia2*^*f/f*^ controls or between selected genotype groups are indicated (***p < 0.001, ****p < 0.0001). **d** Scheme of thalamocortical (green) and corticothalamic axons (red), and immunolabelling with L1- (green) and TAG1-specific antibodies (red) on coronal sections of brains from embryonic day (E) 14.5 mice of the indicated genotypes (DT, dorsal thalamus; VT, ventral thalamus; TE, thalamic eminence; ic, internal capsule). The white arrow points to deviating thalamocortical axons in the *St8sia2*^*−/−*^ embryo. **e** Labeling of perineuronal nets with Wisteria floribunda agglutinin (PNN, red) to visualize the reticular thalamic nucleus (Rt), and neurofilament staining of fibers (NF, green) on coronal sections of brains from P90 mice of the indicated genotypes. Boxed areas highlight higher magnification views in the following column. Scale bars, 1 mm in (**a**) and (**e**), left column, 500 μm in (**d**), 100 μm in (**e**), middle and right column.

Following our previous studies on defective brain connectivity in mice with complete or partial loss of polySia [29, 38], morphometric evaluations of the cKO lines in comparison to *St8sia2*^*f/f*^ and *St8sia2*^*−/−*^ mice were performed at postnatal day 90 (P90). In good agreement with the former results, the rostrocaudal extent of the corpus callosum and the internal capsule were significantly reduced in *St8sia2*^*−/−*^ mice (Fig. 1a–c). As expected, the phenotype of a shorter corpus callosum was fully reproduced in mice with cortical ablation of *St8sia2* (*Emx1-Cre;St8sia2*^*f/f*^ and *Foxb1-Cre;Emx1-Cre;St8sia2*^*f/f*^), while the other cKO lines were unaffected. In contrast, mice with cKO targeting the diencephalon and brainstem (*Foxb1-Cre;St8sia2*^*f/f*^ and *Foxb1-Cre;Emx1-Cre;St8sia2*^*f/f*^) displayed a minor size reduction of the internal capsule, but the strong hypoplasia observed in the *St8sia2*^*−/−*^ mice was not recapitulated in any of the cKO lines. In polySia-negative mice (*St8sia2*^*−/−*^;*St8sia4*^*−/−*^) the smaller internal capsule has been linked to pathfinding defects of thalamocortical and corticothalamic axons during embryonic development [40]. The same pattern of thalamocortical axons that deviate from their normal trajectory without passing through the internal capsule, as well as a prominent loss of corticothalamic axons was detected in E14.5 *St8sia2*^*−/−*^ embryos (Fig. 1d). Unexpectedly, pathfinding and encounter of these axons in *Foxb1-Cre;St8sia2*^*f/f*^, *Emx1-Cre;St8sia2*^*f/f*^ and *Foxb1-Cre;Emx1-Cre;St8sia2*^*f/f*^ at E14.5 was indistinguishable from the wild-type situation in *St8sia2*^*f/f*^ embryos (shown exemplarily for *Foxb1-Cre;Emx1-Cre;St8sia2*^*f/f*^ in Fig. 1d). Consistent with the presence or absence of the embryonic malformations, the previously described disorganized pattern of thalamocortical and corticothalamic axons at the level of the reticular thalamic nucleus [21] was only observed in *St8sia2*^*−/−*^ mice, but not in any of the cKO lines (shown for *Foxb1-Cre;Emx1-Cre;St8sia2*^*f/f*^ in Fig. 1e). In addition, a comparative Western blot analysis of the thalamus-specific vesicular glutamate transporter VGLUT2 in the cortex of *Foxb1-Cre;St8sia2*^*f/f*^ mice yielded no signs of the reduced glutamatergic thalamocortical input observed before in *St8sia2*^*−/−*^ mice [21] (Supplementary Fig. 2). Together, these data indicate that deficits of thalamus-cortex connectivity are caused by loss of *St8sia2* in cells that are not targeted by *Foxb1-* or *Emx1-Cre* driven recombination.

**Fig. 2:**
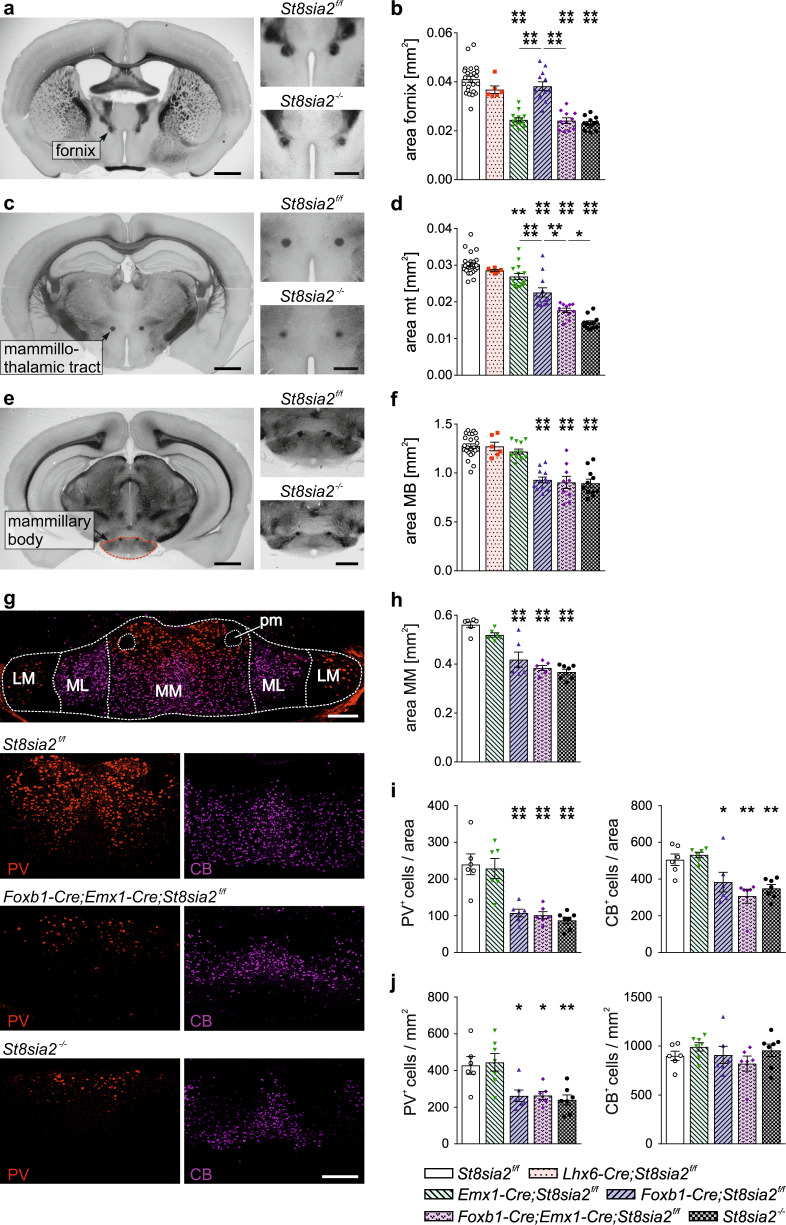
Ablation of *St8sia2* by *Emx1-* or *Foxb1*-*Cre* causes distinct size reductions of fornix, mammillothalamic tract and mammillary body. **a**–**f** Representative images of crossections and evaluations of crossectional areas of the postcommissural fornix at bregma −0.22 mm (**a**, **b**), the mammillothalamic tract (mt) at bregma −1.82 mm (**c**, **d**) and the mammillary body (MB) at bregma ­2.80 mm (**e**, **f**). Scale bars, 1 mm (overviews) and 500 µm (higher magnifications). Graphs show means ± SEM and individual data points of *n* = 24, 6, 14, 12, 11, and 12 (**b**, **d**) or *n* = 24, 6, 12, 12, 9, and 11 (**f**) *St8sia2*^*f/f*^, *Lhx6-Cre;St8sia2*^*f/f*^, *Emx1-Cre;St8sia2*^*f/f*^, *Foxb1-Cre;St8sia2*^*f/f*^, *Foxb1-Cre;Emx1-Cre;St8sia2*^*f/f*^, and *St8sia2*^*−/−*^ mice, respectively. One-way ANOVA indicated significant differences (*p* < 0.0001; F_5,73_ = 44.25 in **b**, F_5,73_ = 66.38 in **d**, F_5,68_ = 29.94 in **f**). Holm–Sidak’s post hoc tests were applied and significant differences for comparisons with *St8sia2*^*f/f*^ controls or between selected genotype groups are indicated (*p < 0.05, **p < 0.01, ***p < 0.001, ****p < 0.0001). **g** Double immunofluorescent staining for parvalbumin (PV, red) and calbindin (CB, magenta) of the mammillary body nuclei on coronal sections of brains from P90 mice at bregma level −0.28 mm. Overview (upper panel) and representative single channel views of the indicated genotypes. MM, medial mammillary nucleus, pars medialis; ML, medial mammillary nucleus, pars lateralis; LM, lateral mammillary nucleus; pm, principal mammillary tract. Scale bars, 200 µm. **h**–**j** Evaluation of the crossectional area of the MM (**h**), the numbers (**i**) and the densities (**j**) of PV- and CB-positive cells in the crossectional area of the MM. Graphs show means ± SEM and individual data points of *n* = 6 *St8sia2*^*f/f*^, *Emx1-Cre;St8sia2*^*f/f*^, *Foxb1-Cre;St8sia2*^*f/f*^, *Foxb1-Cre;Emx1-Cre;St8sia2*^*f/f*^, and *St8sia2*^*−/−*^ mice, respectively. One-way ANOVA indicated significant differences with *p* < 0.0001 for the areas in (**h**) (F_4,26_ = 27.10), for the numbers of PV- and CB-positive neurons in (**i**) (F_4,26_ = 16.20 and F_4,26_ = 8.831), with *p* = 0.0004 for the denisities of PV-positive neurons in (**j**) (F_4,27_ = 7.211), but not for the densities of CB-positive neurons in (**j**) (*p* = 0.42; F_4,27_ = 1.002). Holm–Sidak’s post hoc tests were applied and significant differences for comparisons with *St8sia2*^*f/f*^ controls are indicated (*p < 0.05, **p < 0.01, ****p < 0.0001). All groups with significant differences to the controls were also different from *Emx1-Cre;St8sia2*^*f/f*^ with at least *p* < 0.05 (not shown).

In *St8sia2*^*−/−*^ mice, strong hypoplasia was also detected for the postcommissural fornix and, similar to the situation in mice with a complete loss of polySia [29], for the mt (Fig. 2a–d). The fornix is the major afferent axon tract of the MB (see Supplementary Fig. 3 for a schematic overview of MB connectivity). It emerges from the hippocampal subiculum and, accordingly, the hypoplasia of the postcommissural fornix was recapitulated in the cKO lines with cortical ablation of *St8sia2* (*Emx1-Cre;St8sia2*^*f/f*^ and *Foxb1-Cre;Emx1-Cre;St8sia2*^*f/f*^; Fig. 2b). Along the same lines, the significant reduction of the mt, projecting from the MB to the anterior thalamic nuclei, was reproduced in mice with *St8sia2* ablation in the diencephalon (*Foxb1-Cre;St8sia2*^*f/f*^ and *Foxb1-Cre;Emx1-Cre;St8sia2*^*f/f*^; Fig. 2d). However, the reduction in *Foxb1-Cre;St8sia2*^*f/f*^ mice was less pronounced and a small effect was observed in *Emx1-Cre;St8sia2*^*f/f*^ mice, adding up to a significantly stronger hypoplasia of the mt in mice with a combined ablation of *St8sia2* in the cortex and the diencephalon (*Foxb1-Cre;Emx1-Cre;St8sia2*^*f/f*^). Possibly, reduced innervation of the MB via the fornix affects its efferent projections to the anterior thalamus. This reduced innervation could also affect the integrity of the MB, and/or a loss of MB neurons may cause the hypoplasia of the mt. To address these possibilities, we first assessed the size of the MB by determination of its cross-sectional area on unprocessed vibratome sections, revealing that cKO targeting the diencephalon (*Foxb1-Cre;St8sia2*^*f/f*^ and *Foxb1-Cre;Emx1-Cre;St8sia2*^*f/f*^) caused exactly the same size reduction as the constitutive knockout (*St8sia2*^*−/−*^; Fig. 2e, f). Despite the impact of cortical *St8sia2* deletion on the fornix and its moderate effect on the mt, the size of the MB in *Emx1-Cre;St8sia2*^*f/f*^ mice was not affected. To analyze, if the size reduction of the MB is linked to a loss of neurons, immunostaining of parvalbumin and calbindin was performed, because in contrast to the labeling of, e.g., cortical interneurons, these markers identify two major populations of glutamatergic projection neurons, particularly in the medial MB [41] (Fig. 2g). Since MB connectivity seems unaffected in *Lhx6-Cre;St8sia2*^*f/f*^ mice, this line was not included in this analysis. Evaluations of the immunopositive area, and overall numbers and densities of labeled cells in the pars medialis of the medial mammillary nucleus (MM in Fig. 2g) corroborated the size reduction of the MB (Fig. 2h) and revealed a substantial decrease of both neuronal populations in all three mouse lines with *St8sia2* depletion in the diencephalon, but not in *Emx1-Cre;St8sia2*^*f/f*^ mice with cortical ablation (Fig. 2i). A calculation of cell densities disclosed a specific decrease of the parvalbumin-positive population (Fig. 2j). These results suggest that neuronal loss, together with a corresponding reduction in neuropil, is a major cause for the reduced MB size in response to *St8sia2* deficiency and that the parvalbumin-positive population of glutamatergic MB projection neurons is particularly vulnerable.

**Fig. 3:**
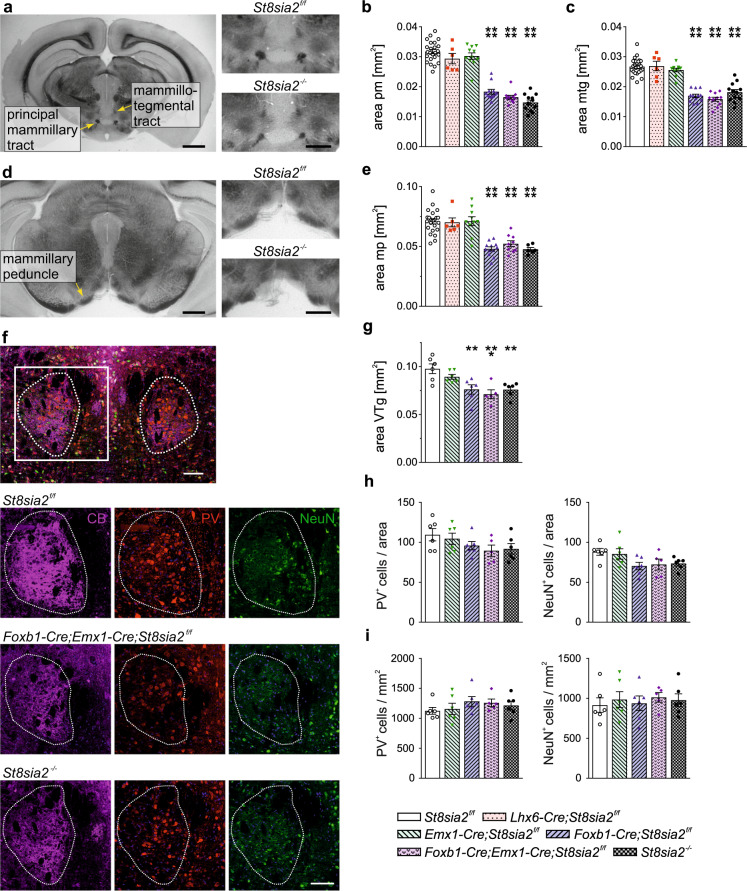
Ablation of *St8sia2* by *Foxb1*-*Cre* affects tegmental connectivity of the MB. **a**–**e** Representative images of crossections (**a**, **d**) and evaluations of crossectional areas of the principle mammillary tract (pm, **b**) and the mammillotegmental tract (mtg, **c**) at bregma −2.70 mm, and the mammillary peduncle (mp) at bregma −3.28 (**e**). Scale bars, 1 mm (overviews) and 500 µm (higher magnifications). Graphs show means ± SEM and individual data points of *n* = 24, 6, 11, 12, 11 and 12 (**b**, **c**) or *n* = 23, 6, 10, 12, 9, and 6 (**e**) *St8sia2*^*f/f*^, *Lhx6-Cre;St8sia2*^*f/f*^, *Emx1-Cre;St8sia2*^*f/f*^, *Foxb1-Cre;St8sia2*^*f/f*^, *Foxb1-Cre;Emx1-Cre;St8sia2*^*f/f*^, and *St8sia2*^*−/−*^ mice, respectively. One-way ANOVA indicated significant differences (*p* < 0.0001; F_5,70_ = 82.54 in **b**, F_5,73_ = 48.90 in **c**, F_5,60_ = 19.24 in **e**). Holm–Sidak’s post hoc tests were applied and significant differences for comparisons with *St8sia2*^*f/f*^ controls are indicated (****p < 0.0001). **f** Triple immunofluorescent staining for calbindin (CB, magenta), parvalbumin (PV, red), and NeuN (green) of the ventral tegmantal nuclei (VTg, dotted outline) on coronal sections of brains from P90 mice at bregma level −4.90 mm. Overview (upper panel) and representative single channel views of the indicated genotypes. Scale bars, 100 µm. **g**–**i** Evaluation of the crossectional area of the VTg (**g**), the numbers (**h**) and the densities (**i**) of PV- and CB-posit**i**ve cells in the crossectional area of the VTg. Graphs show means ± SEM and individual data points of *n* = 5 *Foxb1-Cre;Emx1-Cre;St8sia2*^*f/f*^ mice or *n* = 6 *St8sia2*^*f/f*^, *Emx1-Cre;St8sia2*^*f/f*^, *Foxb1-Cre;St8sia2*^*f/f*^, and *St8sia2*^*−/−*^ mice, respectively. One-way ANOVA indicated significant differences with *p* = 0.0007 for the areas in (**g**) (F_4,24_ = 6.992), but not for the numbers or densities of PV- and CB-positive neurons in (**h**) and (**i**) (*p* = 0.22, F_4,24_ = 1.55 and *p* = 0.059, F_4,24_ = 2.630 for PV and NeuN in **h**; *p* = 0.94, F_4,24_ = 0.19 and *p* = 0.55, F_4,24_ = 0.79 for PV and NeuN in **i**). Holm–Sidak’s post hoc test was applied for data in (**g**) and significant differences for comparisons with *St8sia2*^*f/f*^ controls are indicated (**p < 0.01, ***p < 0.001). When compared to *Emx1-Cre;St8sia2*^*f/f*^, differences with *p* < 0.05 were only observed for *Foxb1-Cre;Emx1-Cre;St8sia2*^*f/f*^.

Based on the differential effects of cortical and diencephalic *St8sia2* ablation on fornix, MB, and mt as central components of the Papez circuit, and motivated by findings on the distinct role of the connectivity between MB and the ventral tegmental nucleus of Gudden (VTg), independent from fornical input [33, 34, 36], we analyzed the components of these connections. As expected, the principal mammillary tract, which is the common efferent pathway of the mammillary body before branching of the axon collaterals that form the mt (see Supplementary Fig. 3), was significantly smaller in *St8sia2*^*−/−*^ mice (Fig. 3a, b), but different from the findings for the mt, this was fully reproduced in cKO targeting the diencephalon (*Foxb1-Cre;St8sia2*^*f/f*^ and *Foxb1-Cre;Emx1-Cre;St8sia2*^*f/f*^). No change was observed in *Emx1-Cre;St8sia2*^*f/f*^, suggesting that the effect of cortical *St8sia2* on the size of the mt is not mediated by the reduced fornical input to the MB. The same was observed for the mammillotegmental tract, formed by the mammillary projections towards the mesencephalic tegmentum after the branching of the mammillothalamic fibers (Fig. 3a, c), and for the mammillary peduncle, consisting mainly of MB afferents originating from the tegmental nuclei of Gudden [42] (Fig. 3d, e). Size and neuron numbers of the VTg were addressed by immunostaining (Fig. 3f). As for the MB, *Lhx6-Cre;St8sia2*^*f/f*^ mice were excluded from the immunohistochemical analysis. Calbindin staining labeled mainly the neuropil and allowed for a precise delineation and evaluation of the VTg area, showing a significant reduction in all genotypes targeting *St8sia2* in the di- and mesencephalon (*St8sia2*^*−/−*^, *Foxb1-Cre;St8sia2*^*f/f*^, and *Foxb1-Cre;Emx1-Cre;St8sia2*^*f/f*^, Fig. 3g). Neuron numbers and densities were analyzed by staining of parvalbumin, a marker of VTg neurons projecting to the MB [43], and the pan-neuronal marker NeuN, resulting in similar patterns of reduced neuron numbers in the genotypes with a smaller VTg (Fig. 3h). However, one-way ANOVA revealed no effect of genotype (*p* = 0.2 and *p* = 0.06 for parvalbumin and NeuN, respectively). Calculation of cell densities also indicated no changes (Fig. 3i). It therefore remained open, if the reduced area of the VTg is caused by a reduced neuropil, the loss of neurons, or, most likely, a combination of both.

In summary, the morphometric analyses revealed that *Foxb1-Cre;St8sia2*^*f/f*^ mice with ablation of *St8sia2* in the di- and mesencephalon show impaired MB connectivity, but no other neuropathological traits of *St8sia2*^*−/−*^ mice.

### Mice with mammillary deficits are hyperactive

**Fig. 4:**
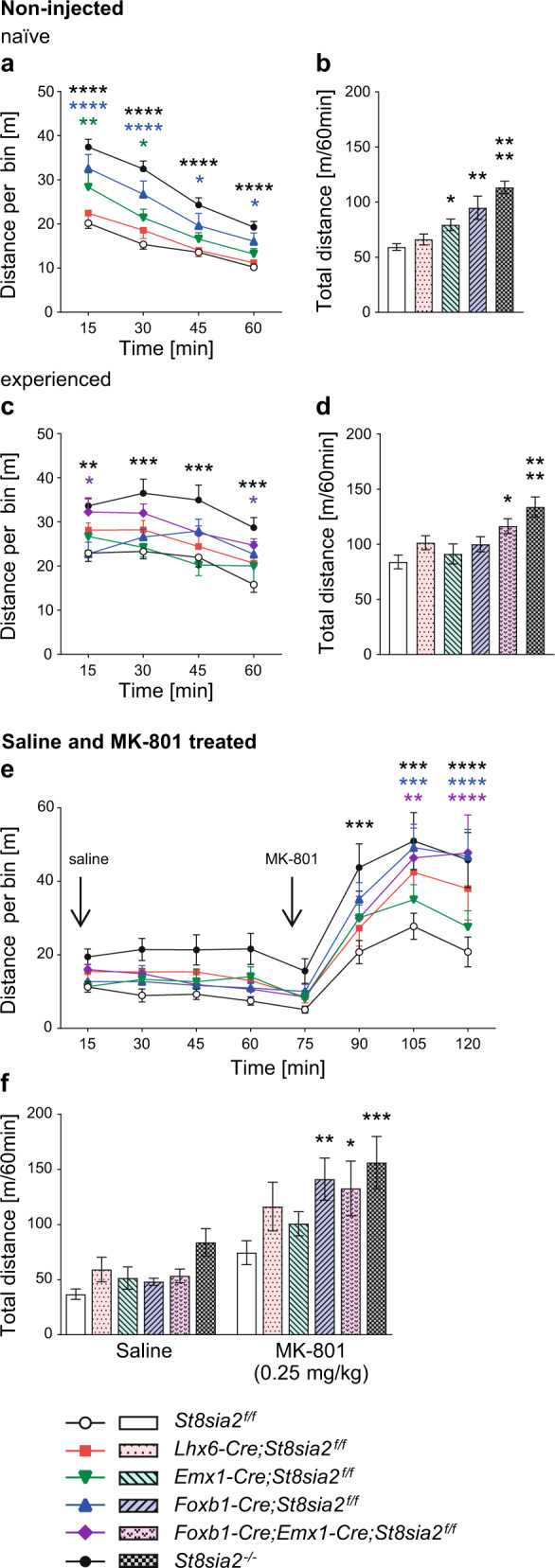
Ablation of *St8sia2* by *Foxb1*-*Cre* increases locomotor activity and susceptibility to MK-801. Activity in the open field arena was monitored over 60 min and evaluated as distance traveled in 15 min bins (line graphs), or as overall distance traveled in 60 min (bar graphs). **a**–**d** Activity of otherwise untreated mice (non-injected) without (naive, cohort 1; **a**, **b**) or with prior experience in the arena (experienced, cohort 2; **c**, **d**; see text for details). **e**, **f** Activity of mice in cohort 2 injected i.p. with saline and MK-801 (0.25 mg/kg body weight) as indicated. Graphs show means and SEM of *n* = 27, 12, 10, 11, and 20 *St8sia2*^*f/f*^, *Lhx6-Cre;St8sia2*^*f/f*^, *Emx1-Cre;St8sia2*^*f/f*^, *Foxb1-Cre;St8sia2*^*f/f*^, and *St8sia2*^*−/−*^ mice in cohort 1 (non-injected, naive; **a**, **b**), and *n* = 12, 10, 5, 16, 14, and 17 or *n* = 16, 12, 14, 18, 14, and 18 *St8sia2*^*f/f*^, *Lhx6-Cre;St8sia2*^*f/f*^, *Emx1-Cre;St8sia2*^*f/f*^, *Foxb1-Cre;St8sia2*^*f/f*^, *Foxb1-Cre;Emx1-Cre;St8sia2*^*f/f*^, and *St8sia2*^−*/−*^ non-injected (experienced; **c**, **d**) or saline and MK-801 treated mice in cohort 2 (**e**, **f**), respectively. Statistics: In **a**, **c**, **e**, and **f**, two-way repeated measure (RM) ANOVA indicated interaction with *p* < 0.0001 (F_12,225_ = 3.67), and differences with *p* < 0.0001 for time (F_3,225_ = 152.4) and genotype (F_4,75_ = 20.13) in (**a**), no significant interaction (*p* = 0.36, F_15,204_ = 1.1), but differences with *p* < 0.0001 for time (F_3,204_ = 12.2), and *p* = 0.0002 for genotype (F_5,68_ = 5.66) in (**c**), interaction with *p* = 0.009 (F_35,602_ = 1.68), and differences with *p* < 0.0001 for time (F_7,602_ = 92.8) and *p* = 0.0091 for genotype (F_5,86_ = 3.29) in (**e**), and no significant interaction (*p* = 0.23, F_5,86_ = 1.4), but differences with *p* < 0.0001 for treatment (F_1,86_ = 78.1), and *p* = 0.009 for genotype (F_5,86_ = 3.29) in (**f**). In **b** and **d**, genotype comparisons indicated differences with *p* < 0.0001 by the Kruskal–Wallis test in **b** (*h* = 42.86) and with *p* = 0.0002 by one-way ANOVA in **d** (F_5,68_ = 5.66). Holm–Sidak’s post hoc tests were applied for data in (**a**) and (**c**–**f**), Dunn’s post hoc test for data in (**b**), and significant differences for comparisons with *St8sia2*^*f/f*^ controls are indicated (*p < 0.05, **p < 0.01, ***p < 0.001, ****p < 0.0001).

A battery of behavioral test was applied to dissect possible contributions of *St8sia2*-dependent deficits in interneuron populations of the prefrontal cortex, reproduced by *Lhx6-Cre;St8sia2*^*f/f*^ mice [20], in cortical connectivity, including corpus callosum and fornix (*Emx1-Cre;St8sia2*^*f/f*^), or in MB connectivity by di- and mesencephalic neurons (*Foxb1-Cre;St8sia2*^*f/f*^). Higher locomotor activity of *St8sia2*^*−/−*^ mice in the open field has been demonstrated before [22, 25] and was reproduced in the two cohorts of mice tested (Fig. 4a–d). Mice in cohort 1 were naive to the open field arena, whereas mice in cohort 2, which included *Foxb1-Cre;Emx1-Cre;St8sia2*^*f/f*^ mice, were experienced because they previously were tested in the dark-light box (see below) in a modified version of the same arena. During the 1 h observation period, naive mice of all genotypes showed a gradual decline in motor activity. Compared to *St8sia2*^*f/f*^ controls, *St8sia2*^*−/−*^ and *Foxb1-Cre;St8sia2*^*f/f*^ mice displayed higher activity over all four, and *Emx1-Cre;St8sia2*^*f/f*^ mice during the first two 15 min bins, resulting in higher overall distances (Fig. 4a, b). In contrast, the higher activity of experienced *St8sia2*^*−/−*^ mice was not mimicked by any of the single cKO lines and only partially reproduced by the *Foxb1-Cre;Emx1-Cre;St8sia2*^*f/f*^ double cKO mice (Fig. 4c, d). The induction of hyperlocomotion by MK-801, a widely used animal model of psychotic behavior [44], was tested with animals of cohort 2 (Fig. 4e, f). Activity levels of all genotype groups remained low after i.p. injection of saline (vehicle) but increased in response to MK-801, and the MK-801-induced hyperlocomotion was strongly enhanced in all genotypes with impaired di- and mesencephalic MB connectivity, i.e., in *St8sia2*^*−/−*^, *Foxb1-Cre;St8sia2*^*f/f*^, and *Foxb1-Cre;Emx1-Cre;St8sia2*^*f/f*^ mice.

### Exacerbated disruption of sensorimotor gating by apomorphine and reduced anxiety in mice with mammillary deficits

**Fig. 5:**
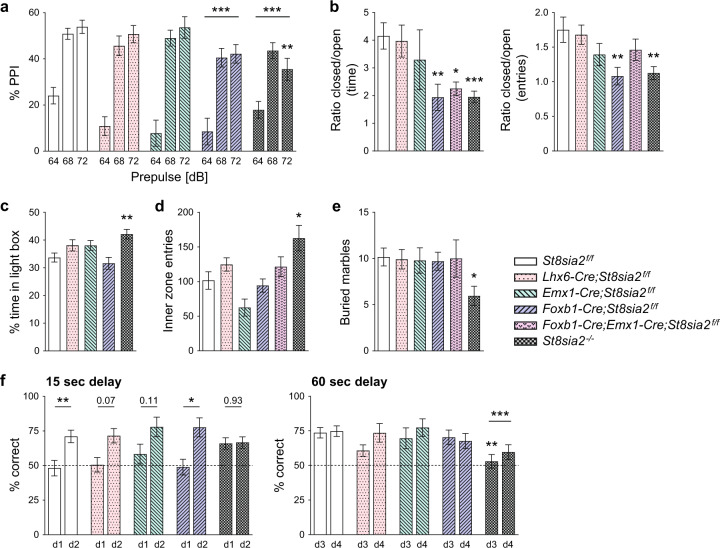
Ablation of *St8sia2* by *Foxb1*-*Cre* impairs PPI and reduces anxiety in the elevated plus maze, but has no effect in other tests of anxiety-like behavior or on working memory. **a** PPI of the acoustic startle response after prepulses of 64, 68, and 72 dB in mice treated with 5 mg/kg apomorphine. Graphs show means ± SEM of *n* = 37, 18, 17, 18, and 27 *St8sia2*^*f/f*^, *Lhx6-Cre;St8sia2*^*f/f*^, *Emx1-Cre;St8sia2*^*f/f*^, *Foxb1-Cre;St8sia2*^*f/f*^, and *St8sia2*^*−/−*^ mice, respectively. **b** Elevated plus maze. Ratios between the time spent in the closed versus the open arm of the elevated plus maze (left), and between the numbers of closed and open arm entries (right). Graphs show means ± SEM of *n* = 15, 23, 6, 17, 5, and 21 (left) or *n* = 16, 24, 6, 19, 6, and 22 (right) *St8sia2*^*f/f*^, *Lhx6-Cre;St8sia2*^*f/f*^, *Emx1-Cre;St8sia2*^*f/f*^, *Foxb1-Cre;St8sia2*^*f/f*^, *Foxb1-Cre;Emx1-Cre;St8sia2*^*f/f*^, and *St8sia2*^*−/−*^ mice, respectively. **c**–**e** Percent of time spent in the light compartment of a dark-light box (**c**), number of entries into the inner zone of the open field (**d**, experienced mice, see Fig. 4c) and number of marbles buried in the marble burying test (**e**). Graphs show means ± SEM of *n* = 16, 20, 17, 15, and 18 *St8sia2*^*f/f*^, *Lhx6-Cre;St8sia2*^*f/f*^, *Emx1-Cre;St8sia2*^*f/f*^, *Foxb1-Cre;St8sia2*^*f/f*^, and *St8sia2*^*−/−*^ mice (**c**), or *n* = 12, 10, 5, 16, 14, and 17 (**d**) and *n* = 23, 24, 18, 23, 9, and 20 (**e**) *St8sia2*^*f/f*^, *Lhx6-Cre;St8sia2*^*f/f*^, *Emx1-Cre;St8sia2*^*f/f*^, *Foxb1-Cre;St8sia2*^*f/f*^, *Foxb1-Cre;Emx1-Cre;St8sia2*^*f/f*^, and *St8sia2*^*−/−*^ mice, respectively. **f** Delayed nonmatch-to-place T-maze task. Percent correct choices (arm alterations) of mice tested on day 1 and 2 (d1, d2) with a forced delay of 15 s (left) and on day 3 and 4 (d3, d4) with a forced delay of 60 s (right). Graphs show means ± SEM of *n* = 32 or *n* = 37 *St8sia2*^*f/f*^ controls for the tests with 15 or 60 s delay, and *n* = 17, 14, 16, and 25 *Lhx6-Cre;St8sia2*^*f/f*^, *Emx1-Cre;St8sia2*^*f/f*^, *Foxb1-Cre;St8sia2*^*f/f*^, and *St8sia2*^*−/−*^ mice, respectively. Statistics: In **a**, two-way ANOVA indicated no significant interaction (*p* = 0.059, F_8,336_ = 1.90), but differences with *p* < 0.0001 for prepulse (F_2,336_ = 105.0) and *p* = 0.0003 for genotype (F_4,336_ = 5.53). In **b**, genotype comparisons for “time” were performed by Welch’s ANOVA after square root transformation, to meet the assumption of normal distribution (left), or by one-way ANOVA (right), indicating differences with *p* = 0.003 (W_5.0,25.13_ = 5.01) or *p* = 0.001 (F_5,87_ = 4.38). In **c**, **d**, and **e**, one-way ANOVA indicated differences with *p* = 0.003 (F_4,81_ = 4.58), *p* = 0.001 (F_5,68_ = 4.60), and *p* = 0.086 (F_5,111_ = 1.99). In **f**, two-way RM ANOVA indicated for the 15 s delay test no significant interaction (*p* = 0.14, F_4,99_ = 1.77), no difference for genotype (*p* = 0.41, F_4,99_ = 1.01), but differences with *p* < 0.0001 for day (F_1,99_ = 22.4), and for the 60 s delay test no significant interaction (*p* = 0.67, F_4,104_ = 0.59), no difference for day (*p* = 0.13, F_4,104_ = 2.27), but differences with *p* = 0.002 for genotype (F_4,104_ = 4.65). In **a** and **f** (right panel), Holm–Sidak’s multiple comparisons tests were used to analyze main effects of genotype. Dunnet’s T3 or Holm–Sidak’s multiple comparisons tests were applied to analyze simple effects of genotype after Welch’s ANOVA in (**b**) (left) or after one- or two-way ANOVA in (**a**), (**b**) (right), and (**c**–**f**). In **f**, Holm–Sidak’s post hoc tests were applied for comparisons between d1 and d2. *p*-values for comparisons between d1 and d2 (**f**, left) or significant differences for comparisons with *St8sia2*^*f/f*^ controls are indicated (*p < 0.05, **p < 0.01, ***p < 0.001).

Altered sensorimotor gating of mice in cohort 1 was assessed by prepulse inhibition (PPI) of the acoustic startle response. Without injection or after i.p. injection of saline (vehicle), none of the *St8sia2*-deficient lines showed significant impairments of PPI at any of the three prepulse intensities tested (Supplementary Fig. 4). After i.p. injection of apomorphine, frequently used to precipitate effects in case of subtle or inconsistent PPI deficits [44], significantly reduced PPI of *St8sia2*^*−/−*^ mice at 72 dB prepulse intensity and a main effect of genotype for *St8sia2*^*−/−*^ and *Foxb1-Cre;St8sia2*^*f/f*^ mice was observed (Fig. 5a). When tested in the elevated plus maze, *St8sia2*^*−/−*^ mice in cohort 2 showed signs of reduced anxiety that were fully reproduced by *Foxb1-Cre;St8sia2*^*f/f*^ and, for the time spent in the closed relative to the open arm, also by *Foxb1-Cre;Emx1-Cre;St8sia2*^*f/f*^ mice (Fig. 5b). Note that some of the mice tested in the elevated plus maze were younger than 100 days. As shown in Supplementary Fig. 5 for the *St8sia2*^*f/f*^ control group, these mice spent significantly more time in the open arm and entered it more often than the older mice. Therefore, mice younger than 100 days were excluded from analysis. However, in other tests of anxiety-like behavior, such as the time spent in the light compartment of a dark-light box (Fig. 5c), the entries into the inner zone of the open field (Fig. 5d), or the number of marbles buried (Fig. 5e), only *St8sia2*^*−/−*^ mice and none of the cKO lines were affected.

Likewise, only the *St8sia2*^*−/−*^ mice displayed signs of impaired spatial working memory in a delayed nonmatch-to-place T-maze task. Mice of cohort 1 were first tested on 2 consecutive days with a 15 s period of forced delay between the sample and the choice run (day 1 and day 2) and, at least 1 week later, with a 60 s delay period (day 3 and day 4; Fig. 5f). These conditions were chosen because a pilot experiment with a small cohort of *St8sia2*^*f/f*^ mice demonstrated an increase of correct choices only during the first 2 out of 4 days of consecutive testing (Supplementary Fig. 6). Similarly, a previous study found that, after a first block of testing for 2 consecutive days, the performance of wild-type and *St8sia2*^*−/−*^ mice remained unchanged over four more blocks [21]. Moreover, only weak effects of *St8sia2* deficiency were detected in this study using a 15 s delay period. In contrast, a more challenging T-maze task with 60 s, but not with 10 s of forced delay yielded significant effects in a study on working memory deficits caused by optogenetic manipulations of thalamo–prefrontal circuit function [45]. For the testing with 15 s delay, performance of all but the *St8sia2*^*−/−*^ group increased above chance level on day 2, resulting in a significant main effect of day, with no effect of genotype (Fig. 5f, left panel, see legend for details on statistics). In *St8sia2*^*−/−*^ mice performance between day 1 and day 2 was unchanged and unlike in the other genotype groups, it was already above chance level on day 1. This unexpected finding may be linked to the different activity and/or anxiety levels of these mice, which may lead to a higher exploratory drive as evident, e.g., by their higher number of inner zone entries in the open field (see Fig. 5d). When tested with a 60 s delay period on day 3 and day 4, all groups started above chance level, as expected based on their previous experience, and no main effect of day was observed. However, there was a significant main effect of genotype, and the *St8sia2*^*−/−*^ mice performed worse, particularly on day 3 (Fig. 5f, right panel).

## Discussion

Here we show that major neuropathological and behavioral traits of *St8sia2*^*−/−*^ mice can be dissected and recapitulated by cKO of *St8sia2*. The di- and mesencephalic ablation of *St8sia2* driven by *Foxb1-Cre* causes compromised MB connectivity and segregates hyperactivity in the open field, deficits of sensorimotor gating, and reduced anxiety in the elevated plus maze from other psychotic and cognitive symptoms of *St8sia2* knockout mice. Based on the numerous associations between genetic variation in *ST8SIA2* and psychiatric disorders with a neurodevelopmental component, we therefore propose that impaired long-range connectivity of the MB has the potential to contribute to these disorders as a result of genetic and neurodevelopmental predispositions.

Importantly, the combination of mammillothalamic and mammillotegmental deficits is the only brain morphological phenotype of *St8sia2*^*−/−*^ reproduced by *Foxb1-Cre;St8sia2*^*f/f*^ mice, because we could exclude disturbed thalamocortical connectivity, which is a major feature of *St8sia2*^*−/−*^ and of completely polySia-negative *St8sia2*^*−/−*^;*St8sia4*^*−/−*^ mice [21, 40]. It remains unclear, why thalamus-cortex connectivity was not affected by single or combined cKO of *St8sia2* in the diencephalon and the cortex. One possible explanation is a delayed onset of polySia reduction after cKO of *St8sia2*, i.e., polySia on thalamocortical and/or corticofugal axons might only be reduced after a developmental event for which the presence of polySia is critical. Another possibility would be that polySia-reductions on cells that are derived neither from the diencephalon nor from the cortex leads to the pathfinding defects observed in *St8sia2* knockout mice. It is also worthy to note that even the complete loss of polySia has no effect on other axon tracts of the diencephalon, such as the optic tract, the posterior commissure or the fasciculus retroflexus and that the development of the mesencephalic dopamine systems is completely normal in *St8sia2*^*−/−*^;*St8sia4*^*−/−*^ mice [29, 46]. Together, this shows that the neurodevelopmental impact of polySia is narrowly confined to specific brain structures, and because other deficits of *St8sia2*^*−/−*^ mice, such as the shorter corpus callosum (current study) and the loss of interneurons in the prefrontal cortex [20] were restricted to mice with cortex- or interneuron-specific loss of *St8sia2*, it can be concluded that the mammillary deficits cause the altered behaviors that were congruently observed in *St8sia2*^*−/−*^ and *Foxb1-Cre;St8sia2*^*f/f*^ mice. In particular, the higher sensitivity of these mice to MK-801, a widely used psychotropic drug to induce schizophrenia-like symptoms in animal models [44], supports the idea that the hypoplasia of the MB and its tegmental connections can cause psychosis-like behavior.

So far, a major experimental approach to study MB functions was analyzing behavioral consequences of lesions. Early studies in the 1970s have established that lesions of the MB, and specifically of its medial part, cause hyperactivity in the open field [30, 31]. Complementary, a more recent study demonstrated that lesions of the VTg, which is reciprocally connected with the medial MB, also result in a significantly higher locomotor activity [33]. Furthermore, reduced anxiety-like behavior in the elevated plus maze has been described after MB lesions [35]. These effects are highly consistent with our current findings and support the assumption that impaired connectivity between MB and VTg causes the observed behavioral deficits in mice with more subtle, genetically induced MB defects.

To the best of our knowledge altered sensorimotor gating has so far not been associated with mammillary deficits, whereas impaired spatial working memory has been frequently reported after genetic MB ablation [32] or in lesion studies targeting MB, VTg or their connectivity [33, 34, 36]. Similarly, chronic amnesia in patients with Wernicke-Korsakoff syndrome has been linked to atrophy of the MB and reduced mammillothalamic connectivity [47, 48]. Regarding the widely assumed mnemonic functions of the Papez circuit [49], the contribution of hippocampal MB input to memory functions appeared self-evident, but this view has been challenged by showing that only mt and VTg lesions, but not lesions of the postcommissural fornix caused impaired spatial working memory [34]. In any case, it was surprising that *St8sia2* cKO mice, reproducing *St8sia2*^*−/−*^ mice in terms of hypoplasia of the postcommissural fornix (*Emx1-Cre;St8sia2*^*f/f*^) or mammillothalamic/mammillotegmental MB connectivity (*Foxb1-Cre;St8sia2*^*f/f*^), did not recapitulate the working memory deficits of *St8sia2*^*−/−*^ mice in the T-maze task. Likewise, despite the firmly established links to parvalbumin-positive interneurons of the prefrontal cortex [50], the working memory deficits of *St8sia2*^*−/−*^ mice were also not reproduced by *Lhx6-Cre;St8sia2*^*f/f*^ mice, although both lines show the same reduction of this interneuron population [20]. Therefore, the working memory deficits of *St8sia2*^*−/−*^ mice must have other causes. As speculated before [21], impaired thalamocortical connectivity seems a plausible cause, as it was observed in *St8sia2*^*−/−*^ but not in any of the cKO lines investigated in the current study. Especially, reduced mediodorsal thalamic projections to the prefrontal cortex could be the underlying neuropathological phenotype, possibly in combination with deficits of parvalbumin interneurons, which are among their targets [45, 51].

A major finding of the current study was the prominent reduction of the parvalbumin-positive projection neurons in the medial MB of mice with di- and mesencephalic ablation of *St8sia2*. The proposed link between deficits of their glutamatergic mammillothalamic and mammillotegmental projections and selected behavioral traits is complementary to a previous study segregating the neurodevelopmental basis of aggressive behavior in *St8sia2*^*−/−*^ mice [23]. Local silencing of *St8sia2* in the early postnatal amygdala caused glutamatergic deficits and aggressive behavior that both could be rescued by local application of the partial NMDA-receptor agonist D-cycloserine. In contrast, local silencing in the amygdala had no effect on anxiety traits, as shown by testing on the elevated plus maze, and intraventricular but not local application of D-cycloserine was efficient in normalizing anxiety levels. It therefore seems reasonable to assume that glutamatergic deficits of MB connectivity in mice with conventional or cKO of *St8sia2* are linked to their reduced anxiety-like behavior in the elevated plus maze.

So far, only few studies have addressed changes in the MB and its connectivity in psychiatric disorders. In schizophrenic patients, postmortem findings on MB volume are conflicting, but reminiscent to the situation in *St8sia2*-deficient mice, reduced neuronal densities and a more than 50% reduction of the parvalbumin-immunoreactive MB projection neurons have been described [52, 53]. MB volumes were reduced in patients with major depression and bipolar disorder [54], whereas no morphometric abnormalities of the fornix were found in patients with schizophrenia, bipolar disorder, and depression [55].

In conclusion, the presented data indicate that impaired MB connectivity is a specific neuropathological trait of mice with a loss of *St8sia2* gene function in the di- and mesencephalon. Especially the increased locomotor activity of these mice, one of the most prominent findings in animal models of psychiatric disorders, and their higher sensitivity to MK-801, a widely used drug to induce schizophrenia-like symptoms [44], support the idea that the hypoplasia of the MB and its tegmental connections can cause psychotic-like behavior, independent from hippocampal input. Hence, the relationship between mammillary body connectivity and behavioral traits should receive more attention in studies on animal models of psychotic behavior. The developmental profile of *St8sia2* expression, its impact on polySia synthesis, and the consequences of its loss consistently indicate that ST8SIA2 exerts its major functions during brain development [19–23, 56–59]. Therefore, our findings provide a plausible link between a genetic predisposition by variation in *ST8SIA2* and neurodevelopmental psychiatric disorders. Future studies should address this possibility, e.g., by advanced brain imaging methods [60, 61] or postmortem analyses of patients with neurodevelopmental psychiatric disorders that have been associated with genetic variation in *ST8SIA2*.

## Supplementary information

Supplementary Material
