# Supplementary material for: Compromised mammillary body connectivity and psychotic symptoms in mice with di- and mesencephalic ablation of ST8SIA2
Source: Transl Psychiatry. 2022 Feb 3;12:51. doi: 10.1038/s41398-022-01816-1 (PMC8814025; doi:10.1038/s41398-022-01816-1)
Supplement: Supplementary file 1 — Supplementary Material [file 41398_2022_1816_MOESM1_ESM.pdf]

Supplementary Material

Compromised mammillary body connectivity and psychotic symptoms in mice with di- and mesencephalic ablation of ST8SIA2

Melike Küçükerden[1,2,#], Ute Schuster[1,#], Iris Röckle[1], Gonzalo Alvarez-Bolado[3], Kerstin Schwabe[2,4], Herbert Hildebrandt[1,2,*]

Affiliations:

[1] Institute of Clinical Biochemistry, Hannover Medical School, Hannover, Germany

[2] Center for Systems Neuroscience Hannover (ZSN), Hannover, Germany

[3] Institute for Anatomy and Cell Biology, Ruprecht-Karls-University Heidelberg, Heidelberg, Germany

[4] Department of Neurosurgery, Hannover Medical School, Hannover, Germany

[#] equally contributing first authors

[*] **Correspondence**:

hildebrandt.herbert@mh-hannover.de

Institute of Clinical Biochemistry, Hannover Medical School,

Carl-Neuberg-Straße 1, 30625 Hannover, Germany,

Phone: +49 511 532 9808

Contents:

Supplementary Methods	page 2
Supplementary Table 1	page 8
Supplementary Figures 1-6	page 9

Supplementary Methods

Mice: Mice were housed in standard plastic cages with their littermates in groups of 2-5 mice per cage in a temperature and humidity-controlled room, under a 12-hour light/dark cycle. Food and water were provided ad libitum.

Mice carrying homozygous loxP flanked ("floxed") *St8sia2* conditional allele (*St8sia2*$^{f/f}$; generated by Taconic Biosciences GmbH, Cologne, Germany) were cross-bred with mice expressing Cre-recombinase under the *zona pellucida 3 (Zp3)* gene promoter (Jackson Laboratory, Bar Harbor, USA; RRID:IMSR_JAX: 003651)(Lewandoski et al., 1997; de Vries et al., 2000), to acquire a line with complete loss of enzymatic ST8SIA2 function (*St8sia2*$^{-/-}$). *St8sia2*$^{f/f}$ mice were also cross-bred with lines expressing the Cre recombinase either under the MGE-derived interneuron-specific *Lhx6* promoter(Fragkouli et al., 2009) (RRID:IMSR_JAX:026555), dorsal telencephalon-specific *Emx1* promoter (Jackson Laboratory, RRID:IMSR JAX:005628) targeting Cajal–Retzius cells, glutamatergic neurons, astrocytes and oligodendrocytes, but not GABAergic neurons(Gorski et al., 2002), or the diencephalon- and brainstem-specific *Foxb1 (forkhead b1)* promoter(Zhao et al., 2007). All of these mouse strains were backcrossed with C57BL/6J mice for at least six generations.

Genotyping was performed with genomic ear or tail DNA using following primers: Cond-1b (5'-GAGACAGCAACTAGAGGAATAACA-3') and cond-2 (5'-CCTAGATGGGTTGGTGTT GC-3') for the floxed *St8sia2* allele; cond-1b and cond-4 (5'-ACAGTTAGAACACCAC CTTC-3') for the recombined *St8sia2* allele; iCreF (5'-GAGGGACTACCTCCTGTACC-3'), iCreR (5'-TGCCCAGAGTCATCCTTGGC-3'), ctrl-F (5'-ACTGGGATCTTCGAACTCTTT GG-3'), and Ctrl-R (5'-GATGTTGGGGCACTGCT CATTCA-3') for the *Lhx6-Cre* allele; Foxb1-iCre-1 (5'-CTCGGCATGGACGAGCTG TACAAG-3'), Foxb1-iCre-2 (5'-CACTGG GATGGCGGGCAACGTCTG-3'), and Foxb1-iCre-3 (5'-CATCGCTAGGGAGTACAAGAT GCC-3') for the *Foxb1-Cre* allele; Emx-s (5'-GCGGTCTGGCAGTAAAAACTATC- 3'), Emx-as (5'-GTGAAACAGCATTGCTGTCAC TT-3'), EmxWT-s (5'-AAGGTGTGGTTCC AGAATCG-3'), and EmxWT-as (5'-CTCTCCACC AGAAGGCTGAG-3') for the *Emx1-Cre* allele.

Brain sectioning and morphological assessment: Mice were anaesthetized with 100 mg/kg Ketamine (CP-Pharma, Burgdorf, Germany) and 8 mg/kg Xylazine (Rompun, Bayer Health Care, Leverkusen, Germany) in 0.9 % (w/v) NaCl and perfused transcardially with approximately 5 ml 0.1 M phosphate buffer (PB), followed by approximately 50 ml 4% (w/v) paraformaldehyde (PFA) in 0.1 M PB solution (pH 7.4). The perfused brain was then transferred into a 4% PFA solution for an additional overnight post-fixation. After post-fixation, brains were washed with 0.1 M PB and stored in 0.1 M PB at 4°C. Free-floating 50 µm thick

coronal sections were obtained using a vibrating blade microtome (Leica Biosystems, Nussloch, Germany; RRID:SCR_016495) and stored in 0.1 M PB at 4°C until further processing. Images were acquired using modified darkfield illumination with a SteReo Discovery.V12 stereomicroscope equipped with an AxioCam MRm digital camera and AxioVision software (Carl Zeiss Microscopy, Göttingen, Germany). Bregma levels of each structure of interest were identified using the Paxinos Mouse Brain Atlas(Paxinos and Franklin, 2001). The cross-sectional area measurements for anatomical structures were performed using the polygon contour tool of ZEN 2012 Imaging Software (Zeiss). To cope with the large number of analyzed mice, we analyzed one section per brain at the level showing the maximal extent of the structure within the serial sections.

Immunofluorescence staining and evaluation: Preparation and staining of 50 µm vibratome sections from embryonic brains and staining of axons traversing the reticular thalamic nucleus labelled with Wisteria floribunda agglutinin in brains from three-month-old mice were performed as described before(Schiff et al., 2011; Kröcher et al., 2015).

Sections of three-month-old mice with the mammillary bodies or the ventral tegmental nuclei were selected according to the Paxinos Mouse Brain Atlas (Paxinos and Franklin 2001). All incubations were performed on an orbital shaker. Sections were permeabilized for 15 min with 0.4% v/v Triton X-100 in PBS, pH 7.4, followed by blocking with 10% v/v fetal bovine serum (FBS) in PBS/Triton for 1 h at room temperature. After overnight incubation with primary antibodies in blocking buffer at 4°C, sections were washed three times for 5 min with PBS/Triton, incubated with secondary antibodies for 1 h at room temperature, washed three times for 5 min and mounted in Vectashield mounting medium with DAPI as a nuclear counterstain (Vector laboratories, Cat# H1200).

For staining of the mammillary bodies or the ventral tegmental nuclei, the following monoclonal (mAb) or polyclonal (pAb) primary antibodies and dilutions were used: Rabbit anti-L1 pAb (kind gift from F. Rathjen, Berlin, Germany, 1:1000), Goat anti-parvalbumin (1:5000; Swant, Bellinzona, Switzerland, Cat# PVG-214; RRID:AB_10000345), rabbit anti-calbindin D-28k (1:5000; Swant, Cat# CB38; RRID:AB_10000340), and mouse anti-NeuN, clone A60, biotin-conjugated antibody (1:1000; Millipore Cat# MAB377B; RRID:AB_177621). Secondary antibody mixture consisted of donkey anti-rabbit IgG, Alexa Fluor 647 (1:500; Thermo Fisher Scientific, Cat# A-31573; RRID:AB_2536183), donkey anti-goat IgG, Alexa Fluor 555 (1:500; Thermo Fisher Scientific, Cat# A21432; RRID:AB_2535853), streptavidin Alexa Fluor 488 (1:500; Jackson ImmunoResearch, Cat# 016-540-084; RRID:AB_2337249).

Microscopy, area measurements, and counting of immunofluorescently labeled cells was performed with an Axio Observer.Z1 microscope equipped with an AxioCam MRm digital camera and ZEN 2012 software (Carl Zeiss Microimaging, Göttingen, Germany). ApoTome technology and a 20x Plan-Apochromat objective with a numerical aperture of 0.8 (Zeiss) was used for structured illumination to acquire 1.62 µm (NeuN), 1.78 µm (parvalbumin) or 2.09 µm (calbindin) thick optical sections. ZEN 2012 (Zeiss) tiles module was used to stitch single images. Per animal, the section showing the maximal extent of the structure of interest within a series of 6 stained sections was evaluated after coding. Images acquired with the same settings and image threshold were blinded and randomized before analysis. Areas were assigned using the polygon contour tool, and immunopositive cells were manually counted with the event counting tool of ZEN Imaging Software (Zeiss).

Behavioral testing

Behavioral tests were performed in the Lower Saxony Centre for Biomedical Engineering, Implant Research and Development. Mice were given at least two weeks for acclimation to the new housing facility after being transferred to the center, where the behavioral experiments were performed. Volumes for injections in the experiments were calculated to correspond to 1% of body weight (e.g., a mouse weighing 30 g was given 0.3 ml).

None of the behavioral experiments was performed on the day of the weekly bedding change. For habituation to the experimenter, each mouse was handled for four days prior to any behavioral procedure.

The activity of mice in the open field, elevated plus maze, and dark-light box experiments was recorded by a digital recorder (TVVR41100, Abus; Affing, Germany) at a rate of 29 frames/second with a light-sensitive digital camera. The videos were analyzed using the automated video-tracking software TopScan 2.0 (TopView Analyzing System 2.0; Clever Sys Inc., Reston, VA, USA).

Open-field locomotor activity and MK-801 treatment: For habituation to the environment, mice were transferred to the experiment room half an hour before the start of the experiment. Cohort 1 was tested in 33 cm (L) x 23 cm (W) x 50 cm (H) polypropylene boxes, while cohort 2 was tested in 36 cm (L) x 26 cm (W) x 50 cm (H) cm polyvinyl chloride (PVC) coated boxes. The tests were performed under 300 Lux luminous intensity. In cohort 1, mice were tested without treatment. In cohort 2, mice were additionally tested after i.p. injections of vehicle (0.9 % saline, i.p.) and 0.25 mg/kg body weight of the non-competitive N-methyl-D-aspartate receptor antagonist MK-801 (Cat# M107, Merck, Darmstadt, Germany). Each test run lasted 60 min.

Thigmotaxis, the tendency to remain close to vertical surfaces, was assessed for the test run without treatment in cohort 2 by measuring the entries into the inner zone of the open field arena, starting at a distance of 9 cm from the walls.

Prepulse inhibition of the acoustic startle response: Prepulse inhibition of the acoustic startle response was assessed using three automated Startle Response System chambers (San Diego Instruments, La Jolla, CA, USA). Each mouse was tested either untreated (no injection), after i.p. injection of 0.9% NaCl (saline vehicle), or after i.p. injection of 5 mg/kg body weight of the nonselective dopamine agonist R-(−)-apomorphine hydrochloride hemihydrate (Cat# A4393, Merck; i.p.) with at least seven days in between. After an injection, mice were directly placed into the test chamber. A 60 dB white background noise was presented during the entire testing period. All mice were acclimated to the white noise for 5 min, before receiving five random 105 dB pulses for 20 ms to acquire a stable startle as a baseline. The prepulse inhibition testing phase consisted of six different acoustic stimuli presented in a randomized order: pulse-only (105 dB for 20 ms), no stimulus (only white noise), prepulse only (72 dB, 20 ms), and three different prepulse stimuli (64, 68, and 72 dB, 20 ms each) followed by a pulse (105 dB for 20 ms) 100 ms after the prepulse. Each stimulus type was presented ten times with 20-30 seconds intertrial intervals (ITI). After the testing phase, mice received another five pulse-only stimuli. Startle responses were defined as the peak startle amplitude in response to each stimulus and were averaged over the trial repetitions. PPI was calculated as a percentage using the following equation: [1- (startle response to prepulse + pulse) / startle response to pulse alone)] x 100.

Delayed nonmatch-to-place T-maze task: The T-maze was made out of black plexiglass and consisted of a 89 cm x 12 cm main platform with a start compartment at one end, leading to two 32 cm x 12 cm goal arms perpendicularly positioned to the main platform. The maze was surrounded by 15 cm high walls. The start compartment and the goal arms were separated from the main platform with remotely controlled sliding doors. At the end of each goal arm, 3 cm diameter food containers were placed. The luminous intensity on the maze was 30 Lux.

Before the experiment proper, each mouse underwent two 10-minute habituation sessions on two consecutive days with open doors and sweetened food pellets in the containers. The food containers were smeared with food pellets to prevent that the smell of the food affected the choice. During the experiment, open arms in the sample runs were offered at equal numbers per day and in a pseudorandomized order with the same open arm offered in no more than two consecutive trials. Each mouse received four trials per day for two consecutive days with an intertrial interval of at least 10 min.

Each trial consisted of a sample run with a forced arm choice and a choice run, in which the mouse had access to both arms. In total, eight trials with a 15 sec delay period between the sample and choice run were performed on day 1 and day 2. After a period of at least one week, eight more trials with a 60 sec delay period were performed (day 3 and day 4). If the mouse alternated the arm in the choice run, the trial was rated "correct", if not, it was rated "false". If a mouse did not leave the start compartment in the choice run for 5 min, the trial was scored not valid. Entries were counted, when all four paws had entered the arm.

Elevated plus-maze: The maze was elevated 85 cm above ground and consisted of two opposing, perpendicularly positioned 30 x 5 cm open and closed arms around a 5 × 5 cm central platform. The luminous intensities of the maze's closed, central, and open parts were 10, 15, and 20 Lux. Mice were placed in the central platform of the maze and their activity was recorded for 5 minutes. The maze was wiped with 10% ethanol after each test. The number of arm entries and spent time in each type of arm was calculated.

Dark-light box: A modified dark-light box test was performed in a box consisting of a large and brightly illuminated (500 Lux) and a smaller dark (10 Lux) compartment. The light compartment dimensions were 48 cm (L) x 36 cm (W) x 50 cm (H) while the dark compartment was 24 cm (L) x 36 cm (W) x 50 cm (H). The compartments were connected by a narrow passage. Mice were placed at one of the outer corners of the light compartment, i.e. at a maximal distance from the entrance to the dark compartment. The activity of mice was monitored for 10 minutes. The time a mouse spent in the light compartment was calculated.

Marble burying: 30 cm (L) x 20 (W) cm x 14 (H) cm polycarbonate cages with fitting filter-top covers were used. Twenty marbles were placed evenly spaced on five cm deep fresh, hand-pressed mouse bedding. Marbles weighed 5.7 gram and had a diameter of 15 mm. The luminous intensity was 250 Lux. Mice were placed individually in a cage and left undisturbed for 30 minutes before buried marbles were counted. A marble was scored "buried", when at least two-thirds of its diameter was covered with bedding. Scoring was performed by a blinded observer on coded pictures of the cages taken before and after the test. Between tests, bedding was changed and the marbles were wiped with 50% alcohol.

Statistics: Shapiro-Wilk and Brown-Forsythe tests were performed to assess normality and equality of variances, respectively. If appropriate, two-tailed *t*-tests were applied, or ordinary one-way, two-way, or two-way repeated measure (RM) ANOVA was used and groups were compared by Holm-Sidak's post hoc test, adjusting for multiple comparisons. For the data on locomotor activity of naïve, non-injected mice

(Fig. 4b), normality was not achieved and the non-parametric Kruskal-Wallis test with Dunn's multiple comparisons post hoc test was applied. To meet the assumption of normal distribution, the ROUT method with a false discovery rate of Q = 1% was used to eliminate two outliers from the dark/light box data (Fig. 5c), and square root transformation was performed for statistical analysis of the ratios of the time spent in the closed versus the open arm of the elevated plus maze (Fig. 5b). Since equal variances were not achieved by square root transformation, Welch's ANOVA with Dunnet's T3 multiple comparisons test was used, as indicated in the corresponding figure legend. The critical alpha value was set at 0.05 for all tests.

References

de Vries WN, Binns LT, Fancher KS, Dean J, Moore R, Kemler R, Knowles BB (2000) Expression of Cre recombinase in mouse oocytes: a means to study maternal effect genes. Genesis 26:110-112.

Fragkouli A, van Wijk NV, Lopes R, Kessaris N, Pachnis V (2009) LIM homeodomain transcription factor-dependent specification of bipotential MGE progenitors into cholinergic and GABAergic striatal interneurons. Development 136:3841-3851.

Gorski JA, Talley T, Qiu M, Puelles L, Rubenstein JL, Jones KR (2002) Cortical excitatory neurons and glia, but not GABAergic neurons, are produced in the Emx1-expressing lineage. J Neurosci 22:6309-6314.

Kröcher T, Malinovskaja K, Jürgenson M, Aonurm-Helm A, Zharkovskaya T, Kalda A, Röckle I, Schiff M, Weinhold B, Gerardy-Schahn R, Hildebrandt H, Zharkovsky A (2015) Schizophrenia-like phenotype of polysialyltransferase ST8SIA2-deficient mice. Brain Struct Funct 220:71-83.

Lewandoski M, Wassarman KM, Martin GR (1997) Zp3-cre, a transgenic mouse line for the activation or inactivation of loxP-flanked target genes specifically in the female germ line. Curr Biol 7:148-151.

Paxinos G, Franklin KBJ (2001) The mouse brain in stereotaxic coordinates. San Diego, CA: Academic Press.

Schiff M, Röckle I, Burkhardt H, Weinhold B, Hildebrandt H (2011) Thalamocortical pathfinding defects precede degeneration of the reticular thalamic nucleus in polysialic acid-deficient mice. J Neurosci 31:1302-1312.

Zhao T, Zhou X, Szabo N, Leitges M, Alvarez-Bolado G (2007) Foxb1-driven Cre expression in somites and the neuroepithelium of diencephalon, brainstem, and spinal cord. Genesis 45:781-787.

Supplementary Table 1. Body weights of mice in cohort 2 prior to testing

Genotype	Body weight in gram (mean ± SEM)	n	Difference against *St8sia2*$^{f/f}$ *
St8sia2$^{f/f}$	30.15 ± 0.496	27	
Lhx6-Cre; St8sia2$^{f/f}$	30.33 ± 0.419	24	p = 0.27
Emx1-Cre; St8sia2$^{f/f}$	29.53 ± 0.548	19	p = 0.43
Foxb1-Cre; St8sia2$^{f/f}$	29.36 ± 0.516	22	p = 0.43
Foxb1-Cre; Emx1-Cre; St8sia2$^{f/f}$	28.78 ± 0.766	9	p = 0.35
St8sia2$^{-/-}$	28.30 ± 0.412	20	p = 0.04

***** Holm-Sidak's multiple comparsions test after one-way ANOVA (p = 0.001)

Supplementary Figures

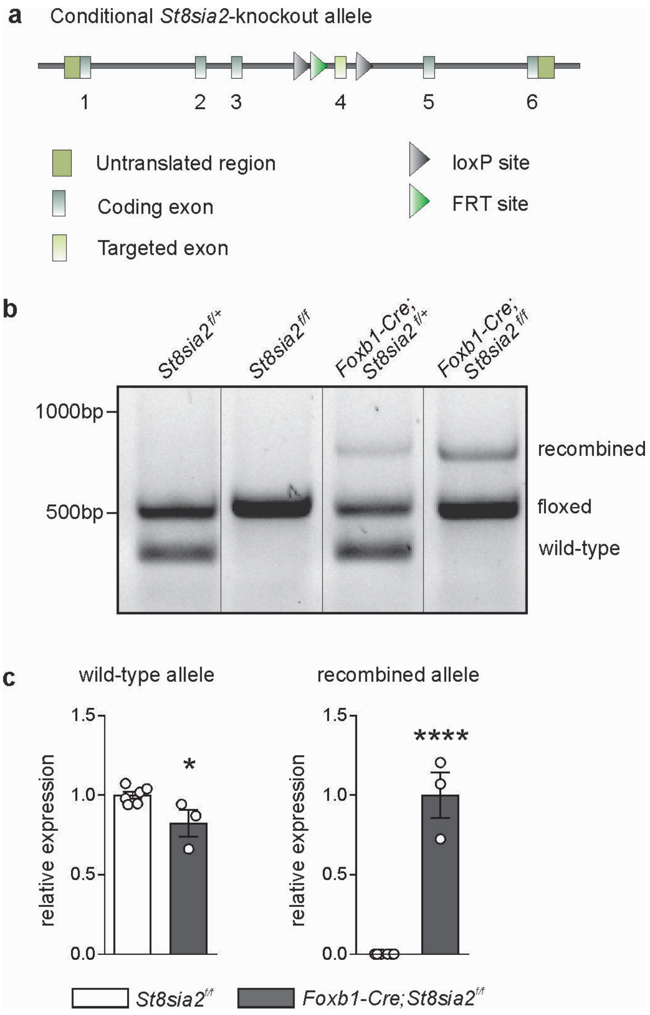

Supplementary Figure 1. Validation of the *Foxb1-Cre* mediated conditional knockout of *St8sia2*. **a** Structure of the conditional *St8sia2* allele with loxp sequences flanking exon 4 (referred to as the 'floxed' allele). **b** Genomic PCR of thalamic tissue isolated from E13.5 embryos homozygous (*f/f*) or heterozygous (*f/+*) for the floxed *St8sia2* allele with or without *Foxb1-Cre* expression, as indicated. **c** qPCR analysis of thalamic tissue isolated from E14.5 $St8sia2^{f/f}$ and $Foxb1\text{-}Cre;St8sia2^{f/f}$ animals detecting transcripts of wild-type (left) and recombined alleles (right) with primers targeting exon 4 of *St8sia2* or the exon 3 to exon 5 boundary obtained by Cre-mediated recombination, respectively. Values for each embryo were obtained by three technical replicates and normalized to the relative expression of wild-type alleles in $St8sia2^{f/f}$ controls (left) or to the relative expression of recombined alleles in Cre-expressing animals (right). Individual values for n=6 wild-type and n=3 knockout embryos and means ± SEM are shown. Statistical analyses by unpaired two-tailed *t*-tests indicated significant differences (wild-type: t_7=2.75, p=0.03; recombined: t_7=10.66, p<0.0001). * p<0.05, **** p<0.0001.

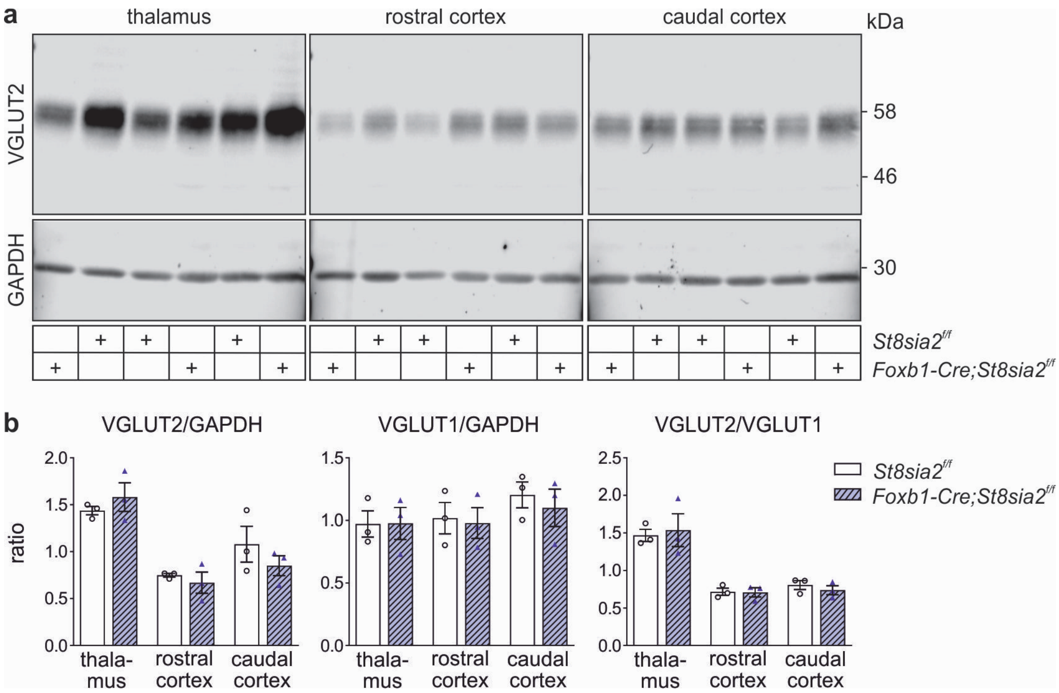

Supplementary Figure 2. Western blot analysis for VGLUT1 and VGLUT2 in thalamic and cortical tissues of P90 mice was performed as described in Kröcher et al. 2015.Thalamus, rostral and caudal cortex samples were acquired from n=3 *St8sia2*$^{f/f}$ and *Foxb1-Cre;St8sia2*$^{f/f}$ mice, each, and measurements performed in four independent Western blot replicates per sample. GAPDH was used as a loading control. **a** Representative Western blot images for VGLUT2 and GAPDH in thalamus, rostral and caudal cortex. **b** Densitometric evaluation. Graphs show means ± SEM and individual data points per animal for VGLUT1 or VGLUT2 signals relative to GAPDH, and for the ratios of VGLUT2 to VGLUT1. Two-way ANOVA indicated no genotype effects.

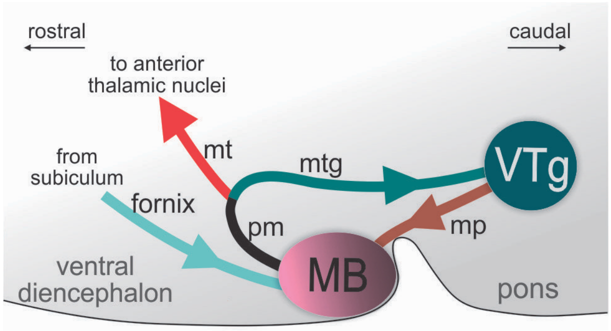

Supplementary Figure 3. Scheme of mammillary body connectivity in a sagittal view. For simplicity, only the reciprocal connections with the ventral tegmental nucleus of Gudden (VTg) are depicted. MB, mammillary body; pm, principal mammillary tract; mtg, mammillotegmental tract; mt, mammillothalamic tract; mp mammillary peduncle.

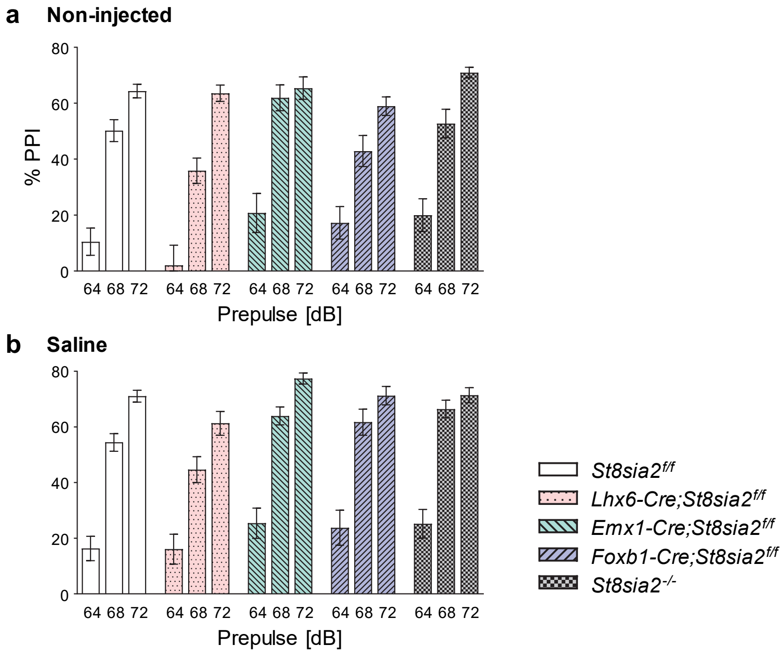

Supplementary Figure 4. Prepulse inhibition (PPI) of the acoustic startle response after prepulses of 64, 68, and 72 dB in otherwise untreated mice (non-injected, **a**) or in mice that received i.p. injections of saline (**b**). Graphs show means ± SEM of n=36-37, 16-18, 16-17, 17-18, and 25-27 *St8sia2*$^{f/f}$, *Lhx6-Cre;St8sia2*$^{f/f}$, *Emx1-Cre;St8sia2*$^{f/f}$, *Foxb1-Cre;St8sia2*$^{f/f}$, and *St8sia2*$^{-/-}$ mice, respectively. To meet the assumption of normal distribution, seven outliers were removed in **a** and two in **b** using the ROUT method. Two-way ANOVA indicated differences with p=0.44 and p=0.72 for interaction ($F_{8,329}$=0.99 and $F_{8,334}$=0.67), p<0.0001 for prepulse ($F_{2,329}$=132.8 and $F_{2,334}$=178.1) and p=0.002 for genotype ($F_{4,329}$=4.27 and $F_{4,334}$=5.64) in **a** and **b** respectively. Holm Sidak's multiple comparisons tests were applied to analyze simple and main effects of genotype for comparisons between the cKO lines and *St8sia2*$^{f/f}$ controls, but no significant differences were detected.

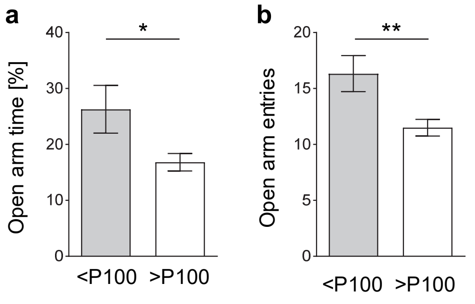

Supplementary Figure 5. Elevated plus maze performance of in *St8sia2*$^{f/f}$ mice younger and older than P100. **a** percent time spent in the open arm. **b** Number of open arm entries. Graphs show means ± SEM of n=6 mice younger, and n=16 mice older than P100. Unpaired two-tailed *t*-tests revealed significant differences (**p*=0.017 in **a**; ***p*=0.005 in **b**).

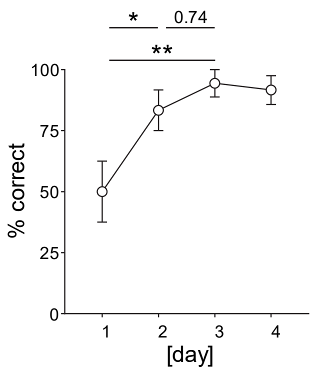

Supplementary Figure 6. Performance of *St8sia2*$^{f/f}$ mice in the delayed nonmatch-to-place T-maze task over four days of consecutive testing with a 15 second delay period. Graph shows means ± SEM of percent correct choices (arm alterations) of n= 9 *St8sia2*$^{f/f}$ mice. One-way ANOVA indicated a significant effect for day with *p*=0.003 ($F_{3,\ 32}$=5.748). Holm-Sidak's post hoc test was applied and *p*-values or significant differences are indicated for selected group comparisons (*p<0.05, **p<0.01).
